# Bio-Orthogonal Nanogels for Multiresponsive Release

**DOI:** 10.1021/acs.biomac.1c00378

**Published:** 2021-06-15

**Authors:** Mohammad Shafee Alkanawati, Marina Machtakova, Katharina Landfester, Héloïse Thérien-Aubin

**Affiliations:** † Max Planck Institute for Polymer Research, Ackermannweg 10, 55128 Mainz, Germany; ‡ Department of Chemistry, Memorial University of Newfoundland, 283 Prince Philip Dr, St. John’s, Newfoundland A1B 3X7, Canada

## Abstract

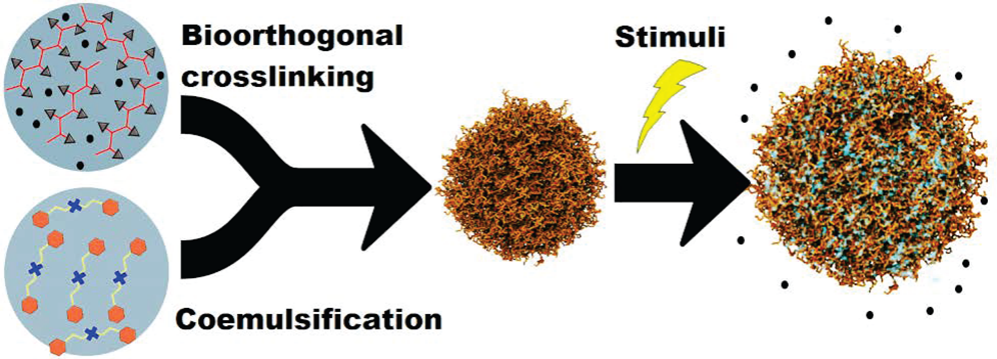

Responsive nanogel systems are interesting for the drug delivery of bioactive molecules due to their high stability in aqueous media. The development of nanogels that are able to respond to biochemical cues and compatible with the encapsulation and the release of large and sensitive payloads remains challenging. Here, multistimuli-responsive nanogels were synthesized using a bio-orthogonal and reversible reaction and were designed for the selective release of encapsulated cargos in a spatiotemporally controlled manner. The nanogels were composed of a functionalized polysaccharide cross-linked with pH-responsive hydrazone linkages. The effect of the pH value of the environment on the nanogels was fully reversible, leading to a reversible control of the release of the payloads and a “stop-and-go” release profile. In addition to the pH-sensitive nature of the hydrazone network, the dextran backbone can be degraded through enzymatic cleavage. Furthermore, the cross-linkers were designed to be responsive to oxidoreductive cues. Disulfide groups, responsive to reducing environments, and thioketal groups, responsive to oxidative environments, were integrated into the nanogel network. The release of model payloads was investigated in response to changes in the pH value of the environment or to the presence of reducing or oxidizing agents.

## Introduction

In recent years, stimuli-responsive nanogels (NGs) have emerged as a class of efficient nanocarriers for drug and gene therapy.^1,2^ Stimuli-responsive NGs combine the properties of other nanocarriers, such as high drug loading, extended biodistribution, and large surface area, allowing for their efficient surface functionalization. Furthermore, using smart hydrogels confers to the NGs the ability to efficiently respond to environmental factors such as temperature, pH, light, magnetic fields, or the presence of certain analytes.^3−8^ In smart hydrogels, the application of a stimulus usually induces modifications in the polymer network through decomposition, isomerization, or supramolecular assembly/disassembly and leads to volume changes between collapsed and swollen states.^9,10^ Additionally, the response to these physicochemical cues can be used to promote the release of active agents encapsulated in the NGs, which make stimuli-responsive NGs a versatile and adaptable class of delivery device to target specific biological abnormalities such as tumor sites where the distinct chemical environment could be used as stimuli.^11^

Stimuli-responsive NGs are especially well suited to develop new chemotherapy treatments. The tumor environment is unique; its characteristic vasculature determines the cellular microenvironment and gives rise to multiple chemical singularities that can be used as stimuli to trigger drug release. For example, in solid tumors, the extracellular pH value can be significantly more acidic (≈5 to 6) than the systemic pH value (7.4) because of the poor vasculature and the resulting anaerobic conditions prevailing in the malignant cells.^12^ Furthermore, certain tumor cells produce reactive oxygen species, including H_2_O_2_, hydroxyl radical, and superoxide, at a higher concentration than healthy cells.^13−15^ Similarly, in other cells, the concentration of glutathione in the blood plasma is 2 μM, and the intracellular glutathione level ranges from 1 to 10 mM in normal tissues; in comparison, the glutathione level in tumor cells can be 7–10-fold larger.^16,17^

Stimuli-responsive NGs can be designed to take advantage of these intrinsic and distinctive properties of the malignant cells to enhance intracellular therapeutic delivery in a tumoral environment. The nonspecific action and poor tumor selectivity sometimes associated with other therapies leading to severe side effects and resistance to chemotherapy could be avoided using carefully designed stimuli-responsive NGs.^18^ For example, the addition of degradable thioketal units that are responsive to the oxidative conditions of the cancerous environment or disulfide linkages responding to a reducing environment or pH-responsive groups can be used in the design of stimuli-responsive NGs to target a specific tumoral environment.^3−5^

Among the methods used to prepare NGs, the gelation of microemulsion precursor droplets is particularly interesting.^19−21^ The main challenge with this method lies in the type of reactions used to form the polymer network, such as heterogeneous free-radical polymerization and unselective chemical cross-linking. In addition to using these potentially harsh chemical conditions, the encapsulation of payloads is often inefficient.^4,22^ To palliate some of those drawbacks, NGs prepared by the self-assembly of polymers through hydrophobic, electrostatic interactions, or hydrogen bonding have been developed. However, their lack of stability after systemic injection can result in their dissociation leading to the premature release of the payload, causing adverse side effects.^23,24^ NGs prepared *via* the cross-linking of miniemulsion droplets can potentially circumvent all of these pitfalls. Using a miniemulsion to prepare NGs allows to tune the diameter of the resulting colloids from nanometers to micrometers and to load large amounts of hydrophilic therapeutic agents.^25^ Additionally, the miniemulsion process is compatible with a wide variety of chemistries, allowing the use of a robust bio-orthogonal gelation process.^26−29^

The gelation reaction leads to the formation of a highly swollen network and can occur through physical or chemical cross-linking. The use of bio-orthogonal chemistry to produce a chemically cross-linked hydrogel is becoming an attractive method to produce new material for the biomedical field. A bio-orthogonal reaction is a reaction that preferably proceeds under normal physiological conditions, does not require the use of toxic catalysts or radiation, has a fast kinetics, does not yield side products, and cannot undergo side reactions with molecules and functional groups present in biological environments.^30^ Such bio-orthogonal chemistries have been used as the cross-linking strategies in the design of new hydrogels.^31,32^ For example, gelatin polymers with pendant tetrazine or norbornene are a pair of reagents that spontaneously undergo bio-orthogonal cross-linking to form hydrogels when they are mixed. Such a system produced injectable gels and maintained the cell-responsive properties of native gelatin.^33^

**Figure 1:**
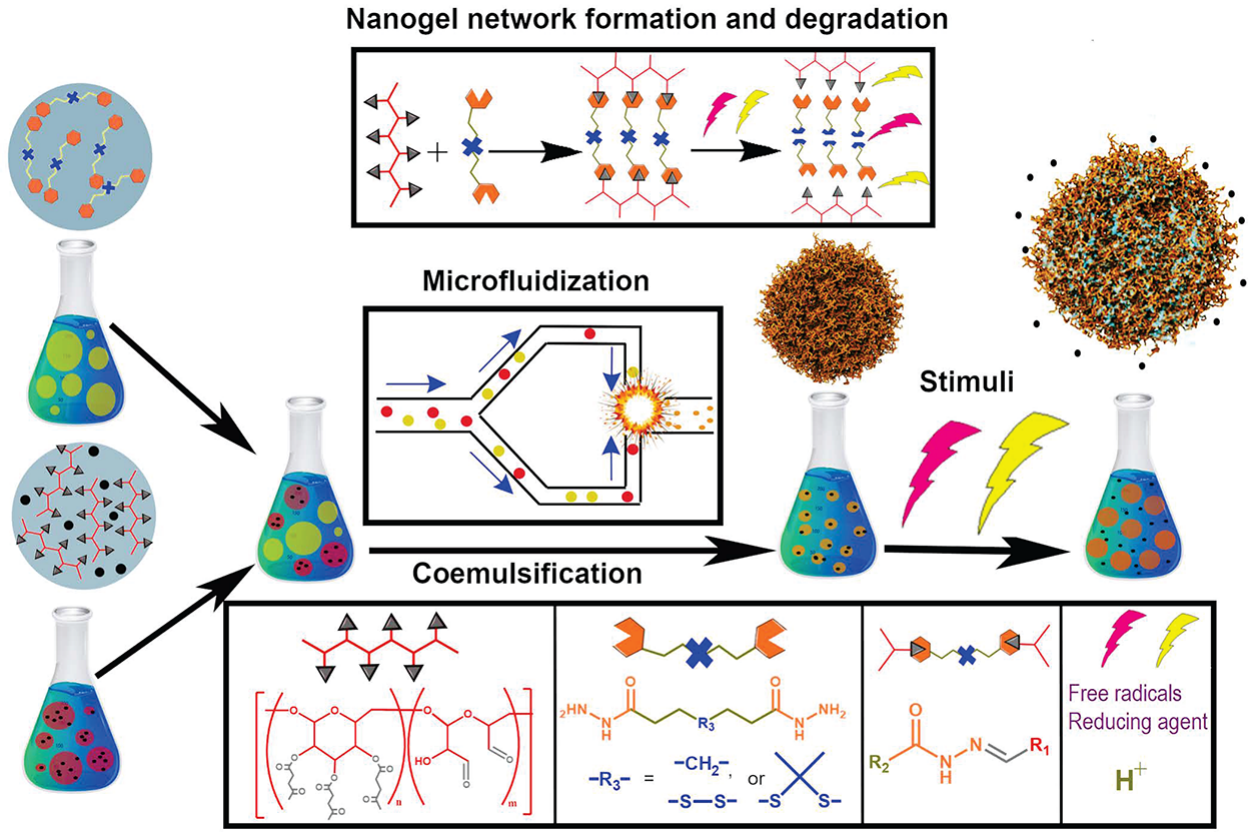
Synthesis of nanogels through coemulsification by microfluidization.

Here, stimuli-responsive NGs were prepared by combining the advantages of the gelation of miniemulsion droplets with robust bio-orthogonal chemistry. The NGs were prepared by the formation of a polyhydrazone network by the cross-linking of nanodroplets of a solution of dextran functionalized with reactive carbonyls and containing a model payload. The reaction occurred when the dextran nanodroplets were combined with nanodroplets of a solution of a responsive hydrazine-functionalized cross-linker (Figure 1). The reaction between the hydrazide and the carbonyl resulted in the formation of a hydrazone network, whose stability is influenced by the pH value of the environment. In addition to its selectivity, this reaction does not need catalysts or harsh conditions, and the liquid precursor droplets were converted in NGs without interfering with the payload and produced no side products that could have a deleterious effect on any biological systems.^34^ Additionally, disulfide and thioketal linkages were built in the cross-linker, leading to the formation of multiresponsive networks. The impact of the pH value and oxidoreducing stresses on the release of the encapsulated species was studied.

## Experimental Section

### Synthesis of the Nanogel Precursors

Functionalized dextran was prepared in a two-step synthesis (see the Supporting Information for the detailed synthesis); first, dextran was oxidized with potassium periodate (KIO_4_) to functionalize the polymer with aldehyde groups,^35,36^ and then the oxidized dextran was reacted with levulinic acid to add ketone groups to dextran (Figure S1). The final polymer was characterized by a combination of titration, NMR and Fourier transform infrared (FTIR) spectroscopy, and gel permeation chromatography (see the SI for details). The resulting polymer had an average molecular weight of 18 kDa and a dispersity index of 2.32 (Figure S2); dextran was functionalized with 0.75 aldehydes/glucose and 0.6 ketones/glucose.

Three cross-linkers were used in the preparation of dextran nanogels. The first one, adipic acid dihydrazide (RNN), was purchased from Sigma-Aldrich, and the others, thioketal dihydrazide (TKNN) and 3,3′-dithiodipropionic acid dihydrazide (DSNN), were synthesized (details are given in the Supporting Information). DSNN was synthesized according to a previously reported method (Figure S3)^37^ by the reaction of hydrazide with a functional diester (diethyl 3,3′-dithiodipropionate). Similarly, TKNN (Figure S4) was prepared by the reaction of hydrazide with a functional diester, dibutyl thioketal dipropionate.

### Synthesis of Nanogels *Via* Coemulsification

Dextran NGs themselves were prepared in an inverse miniemulsion. The miniemulsion process involved the preparation of two separate miniemulsions A and B, where droplets A contained an aqueous solution of a dextran derivative. In contrast, droplets B contained an aqueous solution of a dihydrazide cross-linker. The same continuous phase was used for both miniemulsions and was prepared by dissolving 70 mg of polyglycerol polyricinoleate (PGPR) in 5 g of cyclohexane. The first dispersed phase (A) was prepared by dissolving 100 mg of functionalized dextran in 0.5 mL of phosphate-buffered saline (PBS) (20 mM) followed by the addition of 10 mg of NaCl. If the nanogels were used for *in vitro* studies, a mixture of 5 wt % Cy5-labeled and 95 wt % unlabeled functionalized dextran was used. When a payload (rhodamine derivatives or fluorescein isothiocyanate (FITC)-albumin) was used, 5 mg of this payload was added to the dispersed phase A. The second dispersed phase (B) was prepared by dissolving a 0.15 mM dihydrazide cross-linker in PBS buffer (20 mM, 0.5 mL).

Both dispersed phases were individually added to separate continuous phases and preemulsified with an Ultraturrax for 30 s. Both A and B were individually emulsified by two cycles through a microfluidizer at 896.3 bar. Then, the two miniemulsions A and B were combined and passed through a microfluidizer at 896.3 bar for two more cycles. The resulting suspension was stirred for 24 h at room temperature. The NGs were purified by two cycles of centrifugation (1200*g*) to remove unreacted chemicals and excess surfactant, and quantitative conversion of the precursor nanodroplets into NGs was observed. The NGs in the suspension in cyclohexane were transferred to water by the addition of 1 mL of the organic dispersion to 3 g of a 0.1 wt % aqueous solution of sodium dodecyl sulfate (SDS) in PBS buffer under mild sonication. Then, the samples were stirred in open vials for 24 h at room temperature to evaporate cyclohexane entirely. Finally, the excess SDS was removed using centrifugal concentrators, followed by redispersion in fresh PBS buffer.

### Determination of the Encapsulation Efficiency

The encapsulation efficiency of the payload was determined after every step of the NG purification. Initially, the payload (FITC-albumin) was dissolved in the nanodroplets containing the dextran solution and was encapsulated *in situ* by the cross-linking reaction occurring when the dextran nanodroplets were combined with the cross-linker nanodroplets. Prior to the transfer of NGs to water, when NGs were washed in cyclohexane, the encapsulation efficiency appeared to be quantitative. Albumin, being insoluble in cyclohexane, was not washed away whether encapsulated or not. However, after the transfer of the NGs to water, unencapsulated or poorly encapsulated, albumin could be washed away. To quantify the encapsulation efficiency, the concentration of albumin was measured using fluorescence spectroscopy to record the emission spectra of the fluorescein tag attached to the albumin (λ_ex_ = 488 nm, λ_em_ = 500–650 nm). First, the concentration of FITC-albumin in the unwashed aqueous suspension was measured from the total fluorescence intensity (*I*_t_) of the FITC-albumin in the solution and the FITC-albumin trapped in the NGs. Then, the samples were separated by centrifugal filtration, and the concentration of unencapsulated FITC-albumin and the concentration of FITC-albumin in the solution recovered from the centrifugal filtration was measured (*I*_w_). Finally, the NGs were redispersed following the centrifugal filtration, and the concentration of FITC-albumin in the resuspended samples was measured (*I*_NG_). Systematically, *I*_NG_ + *I*_w_ = *I*_t_, and the encapsulation efficiency was defined as 100(*I*_t_ – *I*_w_)/*I*_t_. The maximal loading was determined by repeating the preparation of the NGs with an increasing amount of FITC-albumin in the dextran nanodroplets (from 0.02 to 1 mg of FITC-albumin per mg of dextran) used to prepare the nanogels.

### Release Kinetics

The release of the payload was measured by fluorescence spectroscopy after the centrifugal ultrafiltration of the NG suspensions incubated for different periods of time in a buffer solution at controlled pH values and concentration of either reducing or oxidizing agents. First, 10–20 mL of the suspension of NGs was concentrated by centrifugal ultrafiltration at 1770*g* using Vivaspin 1000K centrifugal concentrators to yield a suspension of NGs with a concentration of *ca*. 3 mg/mL in phosphate buffer (pH = 7.4). Then, the release was monitored for 3 h in this suspension. After an appropriate period of time (0, 0.5, 1, and 3 h), an aliquot (250 μL) of the NG suspension was filtered by centrifugal ultrafiltration at 1770 *g* for 15 min using a spin filter (Vivaspin 500 μL 1000K). After 3 h of the release, the remaining sample was split into three to five aliquots of 1 mL each. Then, the aliquot was diluted by the addition of 2 mL of a solution at an appropriate pH value and concentration of the oxidizing or the reducing agent to yield final suspensions of a concentration of *ca*. 1 mg/mL of NGs. Then, after appropriate time intervals, an aliquot (250 μL) of the NG suspension was neutralized and filtered by centrifugal ultrafiltration at 1770*g* for 15 min using a centrifugal concentrator. The concentration of the payload released was measured from the fluorescence intensity of the filtrate. The fluorescence of the samples containing FITC-albumin was measured with λ_ex_ = 492 nm and λ_em_ = 518 nm. Due to the quenching of fluorescence caused by the presence of H_2_O_2_, the concentration of the protein released in these samples was measured using a Brandford protein kit (Sigma-Aldrich).

### Cell Viability and Cellular Nanocarrier Uptake

The viability of the cells was quantified with the CellTiter-Glo luminescent cell viability assay (Promega, Germany) after coincubation of the cells with the nanogel suspension with a concentration ranging from 37.5 to 150 μg/mL for 2 and 24 h. The uptake of the NGs by HeLa cells was quantified by flow cytometry after the coincubation of the cells with a suspension of 75 μg/mL for 2 and 24 h. Details of the *in vitro* experiments are described in the Supporting Information.

## Results and Discussion

The formation of NGs resulted from the reaction between aqueous miniemulsion droplets containing the functionalized dextran and the other aqueous miniemulsion droplets containing the water-soluble cross-linker. To accelerate the mixing between the two populations of droplets, equivalent volumes of the two miniemulsions containing an equimolar amount of reactive groups were combined and passed through a microfluidizer to provoke and facilitate the collision and mixing between complementary droplets. To quantify the efficiency of the mixing in the microfluidizer, a model system where the aqueous phase A contained a solution of rhodamine and fluorescein in dilute HCl (0.01 M) and the aqueous phase B contained a solution of NaOH (0.01 M) was used. Upon mixing, the pH of the solution of fluorescent probes was neutralized, and since the signal from fluorescein decreases in acidic media, the variation of the relative fluorescence of rhodamine and fluorescein was used to monitor the pH (Figure S5). The results show that after two to three cycles in the microfluidizer, the two initially distinct aqueous phases were thoroughly mixed.

An aqueous suspension of modified dextran and an aqueous suspension of different cross-linkers were combined in the microfluidizer to prepare NGs. Dextran was functionalized with aldehyde and ketones and bore 0.75 aldehydes/glucose and 0.6 ketones/glucose. Different molecules bearing two acylhydrazide groups were used as cross-linkers. The first cross-linker used, adipic acid dihydrazide (RNN), was used as a model cross-linker. In bulk, the cross-linking of the functionalized dextran with RNN resulted in the formation of dynamic gels where the hydrazone cross-linking points can be reshuffled, providing the bulk gel with a self-healing ability (Figure S6) and this behavior was largely dependent on the pH value of the environment. In mildly acidic media, the partial hydrolysis of the hydrazone linkages led to a faster self-healing behavior. This property could be harnessed in NGs to endow the NGs with a pH-responsive release.^38^ In addition to the pH-responsive cross-linking points, the cross-linkers were also functionalized with chemical functionalities able to be degraded in response to specific biochemical cues. In addition to RNN, 3,3′-dithiodipropionic acid dihydrazide (DSNN) was prepared to introduce chemical groups sensitive to the presence of a reducing agent like glutathione or dithiothreitol, and thioketal dipropionic acid dihydrazide (TKNN) was prepared to introduce chemical groups sensitive to the presence of an oxidizing agent like hydrogen peroxide. The resulting NGs (Table 1) were labeled DNG_X_, where X is the cross-linker used.

**Table 1:** Characteristics of the Nanogels Prepared

		Z-average hydrodynamic diameter (nm)		PDI		
nanogel	cross-linker	cyclohexane	water	cyclohexane	water	ζ-potential (mV)
DNG_RNN_	RNN	139	245	0.03	0.19	–12 ± 1
DNG_DSNN_	DSNN	132	285	0.07	0.18	–9 ± 1
DNG_TKNN_	TKNN	121	290	0.13	0.22	–11 ± 4
DNG_TKNN,DSNN_	TKNN + DSNN	144	268	0.11	0.19	–13 ± 3

**Figure 2:**
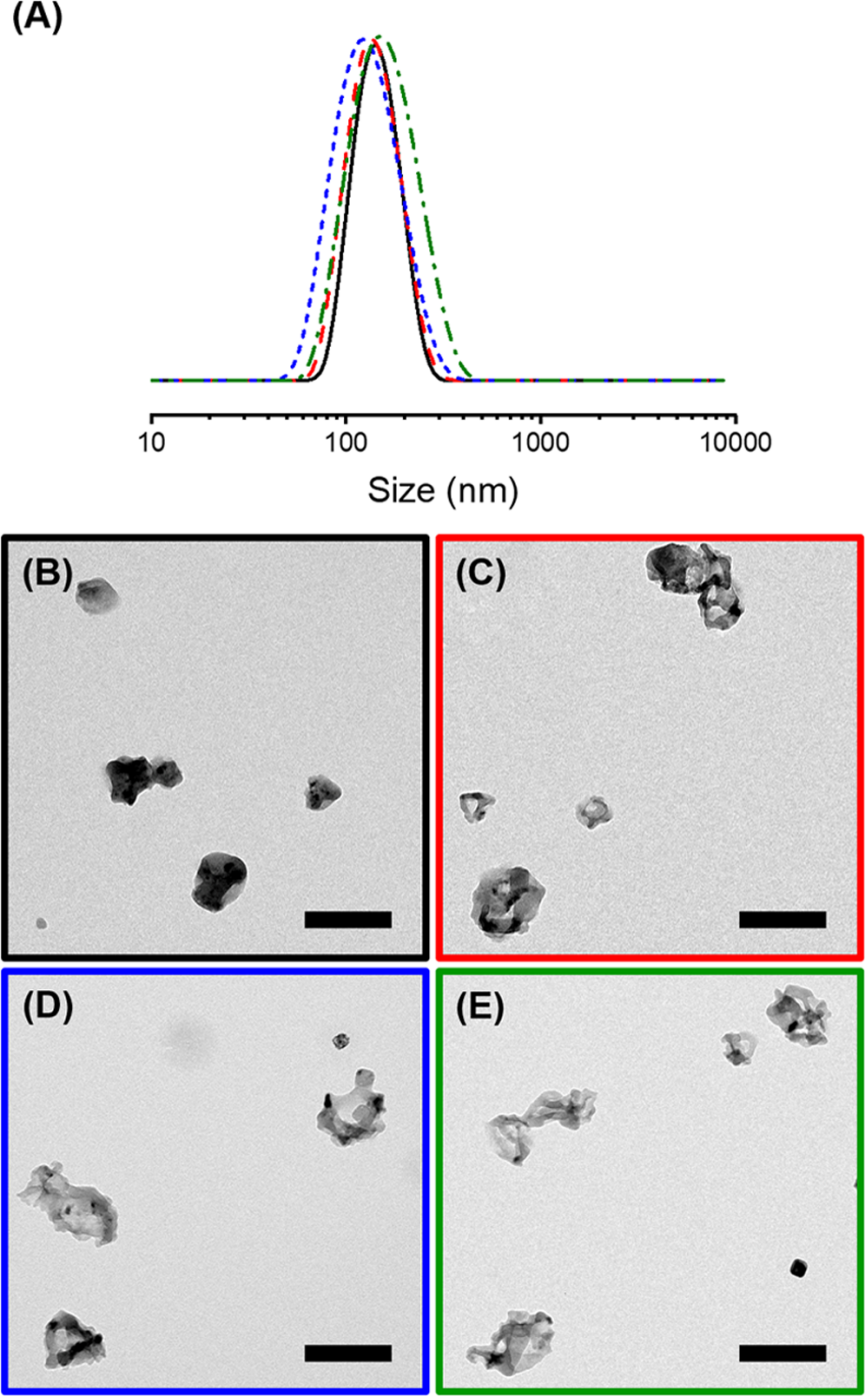
Size distribution (A) and TEM (B–E) images of the dextran nanogels containing FITC-albumin prepared with adipic acid dihydrazide (RNN) (black, B), 3,3′-dithiodipropionic acid dihydrazide (DSNN) (red, C), thioketal dipropionic acid dihydrazide (TKNN) (blue, D), or a mixture of DSNN and TKNN (green, E). The scale bars are 250 nm.

The DNGs obtained were of uniform diameter with a relatively small size distribution (Table 1 and Figure 2). All of the DNGs were prepared with an equimolar amount of hydrazide functionalities and reactive carbonyl groups. The formation of the hydrazone network systematically resulted in the formation of NGs, and the diameter of the resulting nanogels was not affected by the cross-linker used to prepare the nanogels. Upon drying on transmission electron microscopy (TEM) grids (Figure 2) or on solid substrates (Figure S7), the nanogels collapsed during drying and appeared as deflated balls when observed by electron microscopy. The DNGs prepared in a suspension in cyclohexane stabilized with PGPR were transferred to water. Although the pH value inside the DNG nanoenvironment, when the DNGs were dispersed in cyclohexane, was the same as the pH value of the transfer medium (7.4), the DNGs moderately swelled after their solvent transfer due to the swelling of the hydrazone network to reach the equilibrium (Table 1 and Figure S8). During the transfer of the DNGs to water, even though the water-swollen DNGs should be highly stable in water, a moderate aggregation of the DNGs was observed as characterized by an increase in the polydispersity index measured by dynamic light scattering (DLS). This aggregation was likely triggered by the presence of remaining PGPR molecules promoting hydrophobic interaction between the DNGs. Before further analysis of the DNG behavior, the aggregates were removed by filtration, and at least 90% of the DNGs present in the organic suspension were successfully resuspended in the final aqueous suspension of DNGs. To favor the effective redispersion of the DNGs in an aqueous medium, the nanogels were first dispersed in a dilute solution of SDS, which was then removed through centrifugal filtration. However, remaining SDS molecules at the surface of the nanogels were responsible for the moderate negative ζ-potential measured for the purified DNG suspension (Table 1 and Figure S9).

DNGs were used to encapsulate a model payload, FITC-albumin, a protein with a molecular weight of 66 kDa. Albumin was dissolved in an aqueous solution of modified dextran used to prepare the DNGs prior to the cross-linking reaction to encapsulate the payload. The payload was encapsulated *in situ* as the cross-linked network of the DNGs was formed. The encapsulation efficiency was measured after the transfer of the DNGs to water as the fraction of FITC-albumin remaining in the DNGs after the separation of the DNGs from the aqueous media by centrifugal filtration (Figure S10A). The observed encapsulation efficiency was measured after the transfer and equilibration of the DNGs in PBS buffer and accounted for both the unencapsulated payload molecules and those released following the transfer to water of the DNGs (24 h, i.e., the time needed to complete the water transfer process and the complete evaporation of the cyclohexane). The payload was added to the dextran solution prior to cross-linking the dextran-containing droplets, and the encapsulation of the payload in the DNGs occurred as the dextran precursors reacted with the cross-linker molecules. The addition of large amounts of payload to the dextran solution precluded the efficient formation of the cross-linked network, resulting in poor encapsulation efficiency. The encapsulation efficiency was *ca*. 70% for payload loading of less than 0.3 mg of FITC-albumin per mg of nanogels, but decreased significantly for higher loading (Figure S10B). Other macromolecules (Figures S10C and S11) can also be encapsulated and released from the DNGs, but the encapsulation efficiency decreased as the molecular weight of the payload decreased and small molecules were immediately released after the transfer of the DNGs to water (Figure S10C). The encapsulation efficiency of FITC-albumin (*M*_n_ = 66 kDa) and rhodamine-labeled dextran (*M*_n_ = 110 and 500 kDa) in the DNGs reached up to 85%. However, smaller rhodamine-labeled dextrans were encapsulated at less than 30% (Figure S10C). Furthermore, the encapsulation efficiency was not affected by the cross-linker used to prepare the DNGs (Figure S10D).

**Figure 3:**
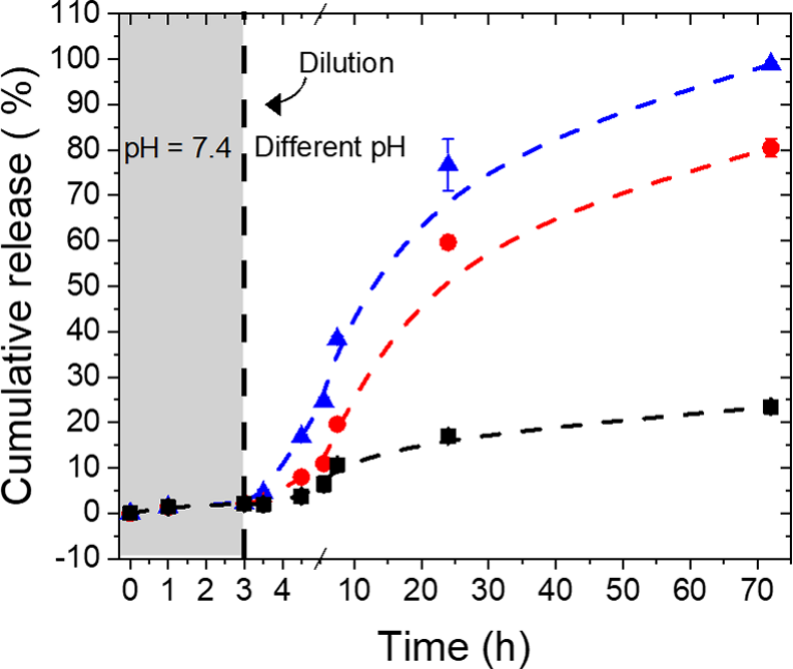
Release of FITC-albumin from DNG_RNN_ in pH 7.4 (■), pH 6.2 (●), and pH 5.2 (▲).

The release of the encapsulated payload was first triggered by changes in the pH value of the DNG environment (Figure 3). When the acidity of the media increased, an increase in the release of the encapsulated protein was observed. After 1 day in a dilute suspension, less than 15% of the protein was released from the DNGs incubated at pH 7.4, but *ca*. 60 and 75% of the encapsulated protein was released in the same period of time after incubation in a buffer at a pH value of 6.2 and 5.2, respectively. The type of reactive carbonyl groups used, ketone or aldehyde, influenced the release kinetic as much as the pH value of the environment.^38^ The hydrazone networks formed by ketone-functionalized dextran were more responsive to changes in acidity than aldehyde-functionalized dextran (Figure S12). These results can be attributed to the higher thermodynamic stability of the acylhydrazone bonds formed between aldehyde and hydrazide in comparison to those formed between ketone and hydrazide.^39,40^ To combine both pH-responsive behavior and the formation of a strong network allowing the successful encapsulation of the payload, both aldehyde and ketone groups were used to functionalize dextran. The hydrazone bonds formed between aldehyde and hydrazide guaranteed the formation of a stable structure of the DNG body, and those formed between ketone and hydrazide provided the pH-responsive behavior. The cumulative release of the cargo from the resulting DNG_RNN_ was slow at neutral pH but increased significantly as the acidity of the suspension increased (Figure 3). This phenomenon occurred due to the dissociation of the acid-sensitive acylhydrazone bonds when the acidity of the environment increased. The same release behavior was observed for all of the DNGs and for different payloads (Figure S10). Such a pH-responsive behavior could be harnessed either to target tumoral environments^12^ or specific cell compartments^41^ that are more acidic than the blood or cytosol.

**Figure 4:**
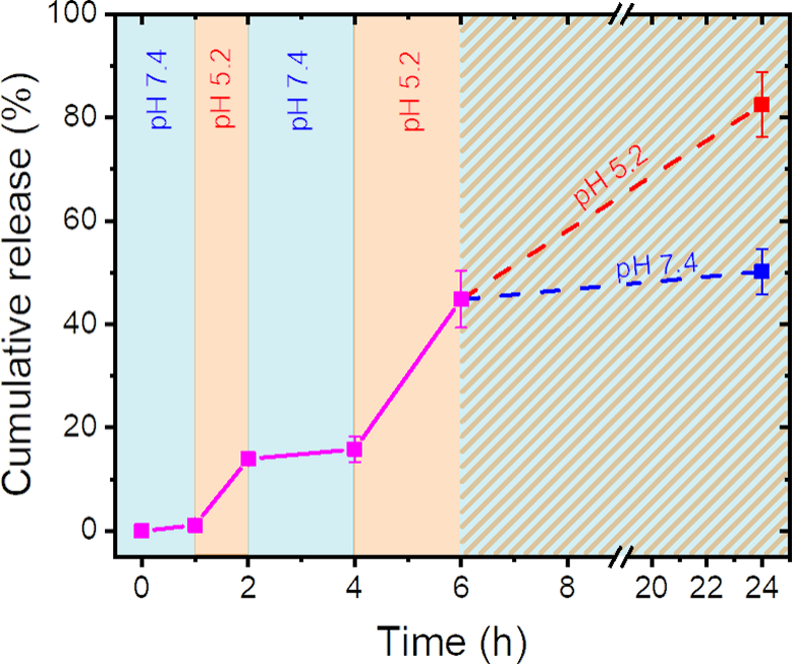
Temporal control of the release of FITC-albumin from DNG_RNN_ by the modulation of the pH value of the environment between 5.2 and 7.4. After 6 h, the sample was divided into two aliquots, one incubated in a suspension at a pH value of 7.4 and the second at a pH value of 5.2.

When DNGs were redispersed from neutral pH buffer to acidic pH buffer, the DNGs swelled by *ca*. 30% (Figure S13). This swelling, triggered by the decrease in the effective cross-linking density, was fully reversible and could be used to switch on and off the release from the DNG suspension (Figure 4). The hydrazone bonds were the cross-linking points of the DNGs and the equilibrium of their formation is affected by the concentration of protons in the solution. Consequently, the variation in the pH value of the environment led to the reversible assembly and disassembly of the hydrazone bonds. Therefore, changing the acidity of the environment of the DNGs by the addition of HCl or NaOH resulted in the stop-and-go release of the payload encapsulated in the DNGs (Figure 4). The results show that a rapid release of the payload was observed at a pH value of 5.2, but slowed down and almost stop at a pH value of 7.4, and the variation of the pH value of the environment could be used to modulate the release profile of the payload from the DNGs.

**Figure 5:**
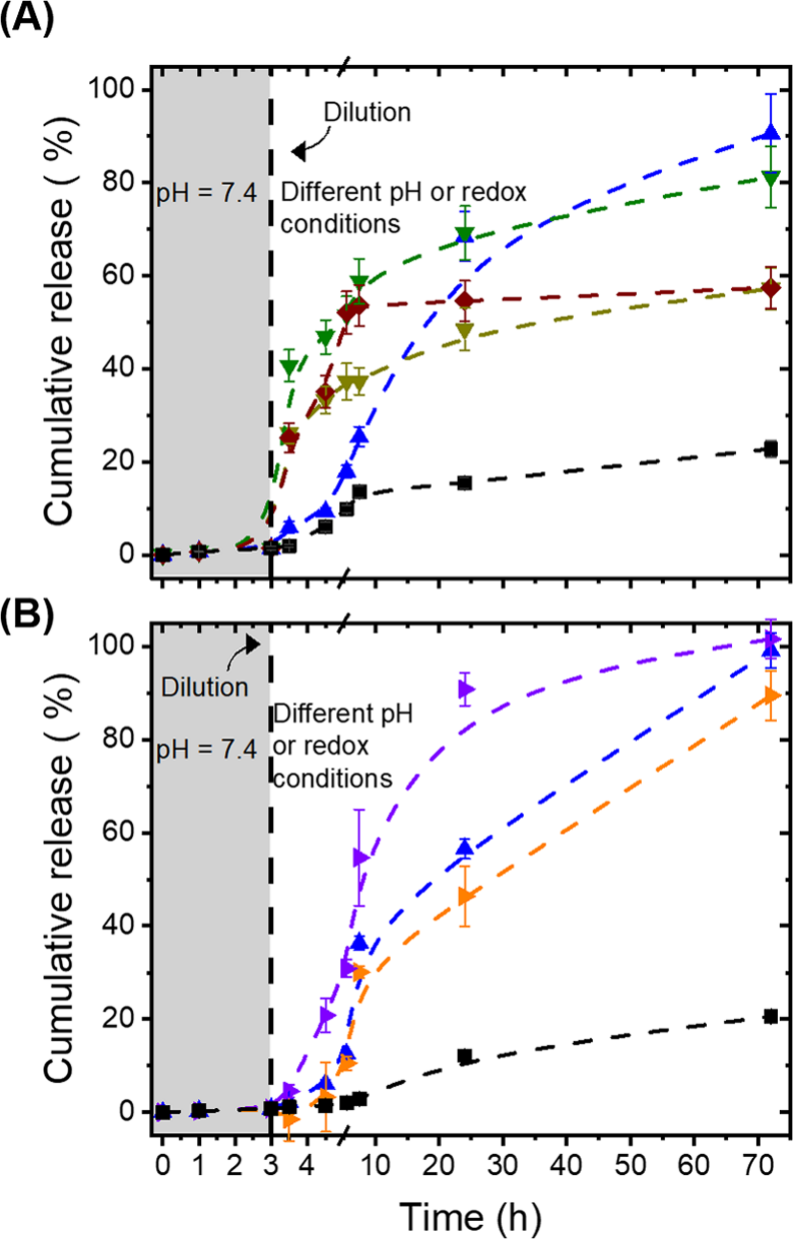
Release of FITC-albumin from (A) DNG_DSNN_ in buffer at (■, black) pH 7.4 or (▲, blue) pH 5.2 and in the presence of a reducing agent (▼, dark yellow) dithiothreitol 50 mM, (▼, green) dithiothreitol 200 mM, and (⧫, burgundy) glutathione 200 mM; (B) DNG_TKNN_ in buffer at (■, black) pH 7.4 or (▲, blue) pH 5.2 or in the presence of hydrogen peroxide (▶, orange) 50 mM or (▶, violet) 200 mM as an oxidant.

To enhance the control over the release kinetic, additional functionalities were added in the design of the cross-linker. Using either DSNN, a disulfide-containing dihydrazide, or TKNN, a thioketal-containing dihydrazide, the release from the DNGs was not only influenced by the pH value of the environment, like in the case of DNG_RNN_, but was also significantly affected by the addition of a reducing or an oxidizing agent (Figure 5). DNG_DSNN_ was sensitive to both changes in the pH value and to the addition of dithiothreitol or glutathione (Figure 5A). In the case of DNG_TKNN_, the presence of H_2_O_2_ led to the release of the payload in addition to variation in the pH value of the environment (Figure 5B). In neutral conditions, all of the DNGs displayed a release of less than 20% of the encapsulated cargo after 24 h of incubation in suspensions at a pH value of 7.4. However, the cargo was released entirely, from every DNGs, when the DNGs were in suspension in acidic media.

Additionally, the DNG cross-linked with DSNN and TKNN preserved the ability to respond to the reducing agent. DNG_DSNN_ released its cargo in the presence of dithiothreitol; up to *ca*. 75% of the encapsulated payload was released after 24 h of incubation with 200 mM dithiothreitol. DNG_DSNN_ was also responsive to the presence of glutathione at a concentration representative of diseased tissue (200 mM). Furthermore, DNG_TkNN_ completely released its payload after incubation with H_2_O_2_ (200 mM), and released *ca*. 65% of the payload after their incubation with H_2_O_2_ (50 mM)

**Figure 6:**
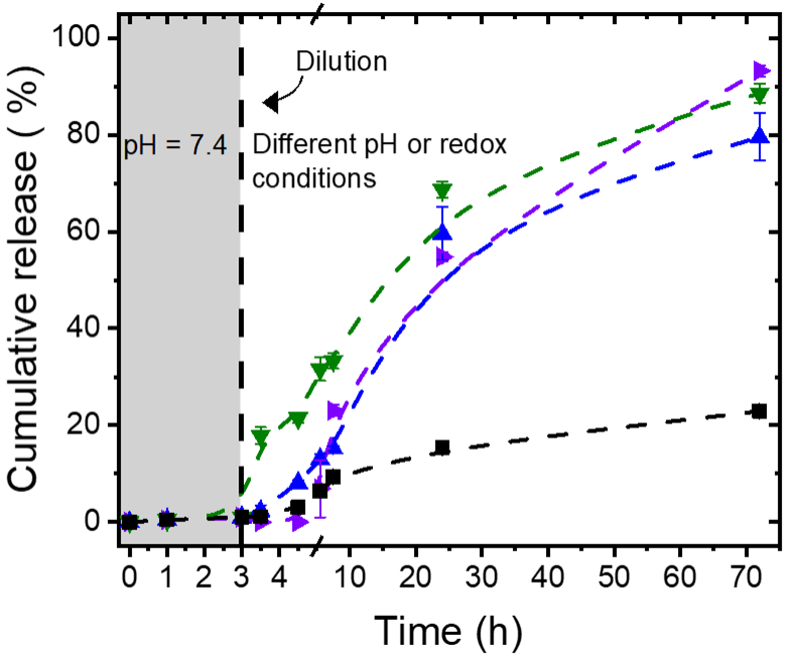
Release of FITC-albumin from DNG_TKNN,DSNN_ in buffer with pH = 7.4 (■) and pH = 5.2 (▲) or in the presence of dithiothreitol 200 mM (▼) or H_2_O_2_ 200 mM (▶).

Interestingly, the DNGs can easily be prepared by combining both DSNN and TKNN in one system. The resulting DNG _TKNN,DSNN_ displayed multistimuli-responsive behavior. Figure 6 shows that DNG_TKNN,DSNN_ did not release the encapsulated albumin when in suspension in a buffer at pH 7.4 (<20% in 24 h), but an efficient release was observed in acidic media and in the presence of dithiothreitol or H_2_O_2_, confirming that DNG_TKNN,DSNN_ can undergo a multiresponsive release.

**Figure 7:**
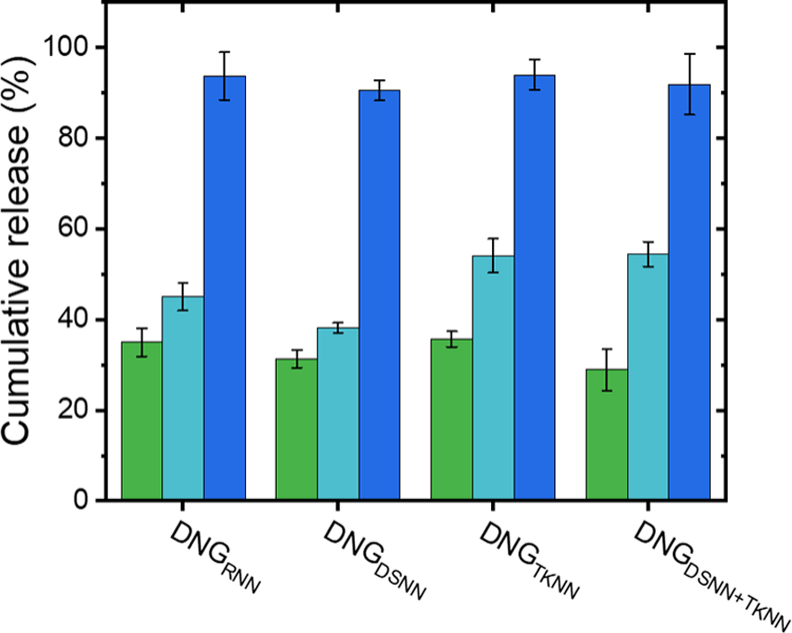
Biodegradability of the nanogels. Release of FITC-albumin from the DNGs prepared with adipic acid dihydrazide (RNN), 3,3′-dithiodipropionic acid dihydrazide (DSNN), thioketal dipropionic acid dihydrazide (TKNN), or a mixture of DSNN and TKNN. The DNGs were incubated at pH = 7.4 with dextranase for 0.5 h (green), 4 h (teal), and 24 h (blue).

The release of the encapsulated FITC-albumin was also studied in the presence of dextranase. Dextranase is an enzyme that hydrolyzes the α-1,6 glycosidic bonds of dextran and is present in the human body in the intestine, kidneys, lungs, and spleen.^42^ Despite the significant chemical modification of the dextran, the DNGs were degraded by the addition of dextranase to the suspensions (Figure 7), resulting in the release of the encapsulated FITC-albumin. The DNG dispersions in PBS buffer (at pH = 7.4) did not show any release over several hours. However, after the addition of dextranase (1 mg of dextranase for 10 mg of DNGs), a fast increase in the fluorescence intensity of FITC-albumin was detected in the supernatant after removing the DNGs by centrifugal filtration. The signal of the released FITC-albumin increased over time as the dextranase degraded the network of the DNGs. The DNGs prepared with different dihydrazide cross-linkers all reacted in a similar manner (Figure 7). The degradation of the nanogels in the presence of dextranase can thus be used as a possible pathway to deliver payloads to dextranase-rich tissues like the colon or the spleen.

**Figure 8:**
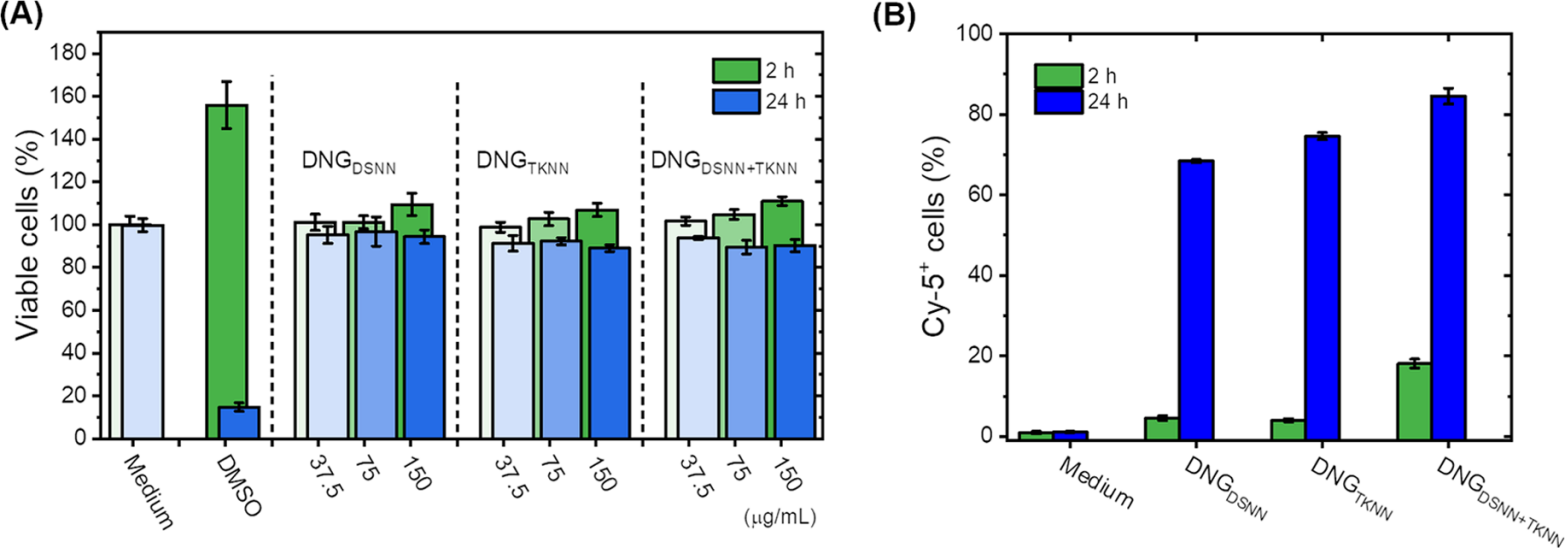
Cytotoxicity (A) and cellular uptake (B) for the dextran nanogels incubated with HeLa cells. (A) Fraction of viable cells was quantified after 2 and 24 h of coincubation of the HeLa cells with increasing concentration of DNGs ranging from 37.5 to 150 μg/mL. (B) Fraction of cells containing the DNGs as evidenced by the presence of cyanine-5 dye was quantified by flow cytometry after 2 and 24 h of coincubation between the HeLa cell and a suspension of DNGs at a concentration of 75 μg/mL.

Finally, the interaction of the dextran nanogels with cells was evaluated after incubation of the DNGs with HeLa cells (Figure 8). The DNGs did not display any cytotoxicity after 1 day of incubation, even at high concentrations. The cellular uptake of the DNGs by HeLa cells was measured using DNGs prepared using functionalized dextran labeled with cyanine-5 (Cy5), a fluorescent tag. The fluorescently labeled functionalized dextran was prepared by the esterification of the remaining alcohol groups on the functionalized dextran with a Cy5-NHS derivative. The resulting fluorescent DNGs were incubated with HeLa cells, and after washing off the free DNGs, the fluorescence of the cells was quantified by flow cytometry. Flow cytometry results showed that the different DNGs were efficiently taken up by the cells. Different DNGs were uptaken in a similar manner by the cells. After 24 h of coincubation, the fraction of the cells that took up DNGs increased in comparison to what was observed after only 2 h of coincubation. This result was indicative of the continuous uptake of the DNGs by the HeLa cells.

## Conclusions

The preparation of multistimuli-responsive nanogels was successful. These nanogels were prepared using a bio-orthogonal reaction between reactive carbonyl and hydrazide groups, leading to the formation of a hydrazone network under biologically relevant conditions. The nanogels were synthesized by performing this cross-linking reaction within the confinement of nanodroplets prepared by a miniemulsion. After the mixing of complementary droplets containing either dextran, bearing the reactive carbonyl groups, or the cross-linker, containing difunctional hydrazide molecules, the cross-linked hydrazone networks were obtained within the nanodroplets. To prepare the nanogels, a dextran precursor was functionalized with both aldehydes and ketone groups on the same polymer chain and resulted in nanogels that displayed high encapsulation efficiency for macromolecular payloads and a pH-responsive release behavior in a physiologically relevant range. The stability of the hydrazone nanogels was influenced by the pH value of the suspensions. An increase in the acidity of the environment led to a disruption of the hydrazone network and the release of the encapsulated payload. Furthermore, the cross-linker dihydrazide molecules were also functionalized with cleavable functionalities to provide alternative release mechanisms. The combination of cross-linker bearing disulfide and thioketal functionalities yielded nanogels able to respond to changes in acidity, the presence of oxidants, or the presence of reducing agents. Given their versatility and their ability to encapsulate and release large macromolecular payloads in simulated biological environments, these nanogels can find application in the development of future therapies involving the delivery of biomacromolecular therapeutic agents such as proteins or oligonucleotides.

## Supporting Information Available

Detailed procedure for the synthesis of the nanogel precursors and additional characterization of the nanogels (PDF)

## References

[ref1] Kandil R.; Merkel O. M. Recent Progress of Polymeric Nanogels for Gene Delivery. Curr. Opin. Colloid Interface Sci. 2019, 39, 11–23. 10.1016/j.cocis.2019.01.005. 30853837 PMC6400264

[ref2] Qu Y.; Chu B.; Wei X.; Lei M.; Hu D.; Zha R.; Zhong L.; Wang M.; Wang F.; Qian Z. Redox/pH Dual-Stimuli Responsive Camptothecin Prodrug Nanogels for “On-Demand” Drug Delivery. J. Controlled Release 2019, 296, 93–106. 10.1016/j.jconrel.2019.01.016. 30664976

[ref3] Shim M. S.; Xia Y. A Reactive Oxygen Species (ROS)-Responsive Polymer for Safe, Efficient, and Targeted Gene Delivery in Cancer Cells. Angew. Chem., Int. Ed. 2013, 52, 6926–6929. 10.1002/anie.201209633. PMC3746021 23716349

[ref4] Yang W. J.; Zhao T.; Zhou P.; Chen S.; Gao Y.; Liang L.; Wang X.; Wang L. “Click” Functionalization of Dual Stimuli-Responsive Polymer Nanocapsules for Drug Delivery Systems. Polym. Chem. 2017, 8, 3056–3065. 10.1039/C7PY00161D.

[ref5] Li Y.; Bui Q. N.; Duy L. T. M.; Yang H. Y.; Lee D. S. One-Step Preparation of pH-Responsive Polymeric Nanogels as Intelligent Drug Delivery Systems for Tumor Therapy. Biomacromolecules 2018, 19, 2062–2070. 10.1021/acs.biomac.8b00195. 29625005

[ref6] Zhou A.; Luo H.; Wang Q.; Chen L.; Zhang T. C.; Tao T. Magnetic Thermoresponsive Ionic Nanogels as Novel Draw Agents in Forward Osmosis. RSC Adv. 2015, 5, 15359–15365. 10.1039/C4RA12102C.

[ref7] Xing Z.; Wang C.; Yan J.; Zhang L.; Li L.; Zha L. Dual Stimuli Responsive Hollow Nanogels with IPN Structure for Temperature Controlling Drug Loading and pH Triggering Drug Release. Soft Matter 2011, 7, 7992–7997. 10.1039/c1sm05925d.

[ref8] Chiang W.-H.; Ho V. T.; Chen H.-H.; Huang W.-C.; Huang Y.-F.; Lin S.-C.; Chern C.-S.; Chiu H.-C. Superparamagnetic Hollow Hybrid Nanogels as a Potential Guidable Vehicle System of Stimuli-Mediated MR Imaging and Multiple Cancer Therapeutics. Langmuir 2013, 29, 6434–6443. 10.1021/la4001957. 23627806

[ref9] Fleige E.; Quadir M. A.; Haag R. Stimuli-Responsive Polymeric Nanocarriers for the Controlled Transport of Active Compounds: Concepts and Applications. Adv. Drug Delivery Rev. 2012, 64, 866–884. 10.1016/j.addr.2012.01.020. 22349241

[ref10] Cabane E.; Zhang X.; Langowska K.; Palivan C. G.; Meier W. Stimuli-Responsive Polymers and Their Applications in Nanomedicine. Biointerphases 2012, 7, 9 10.1007/s13758-011-0009-3. 22589052

[ref11] Mandal P.; Maji S.; Panja S.; Bajpai O. P.; Maiti T. K.; Chattopadhyay S. Magnetic Particle Ornamented Dual Stimuli Responsive Nanogel for Controlled Anticancer Drug Delivery. New J. Chem. 2019, 43, 3026–3037. 10.1039/C8NJ04841J.

[ref12] Vaupel P.; Kallinowski F.; Okunieff P. Blood Flow, Oxygen and Nutrient Supply, and Metabolic Microenvironment of Human Tumors: A Review. Cancer Res. 1989, 49, 6449–6465. 2684393

[ref13] Trachootham D.; Alexandre J.; Huang P. Targeting Cancer Cells by Ros-Mediated Mechanisms: A Radical Therapeutic Approach?. Nat. Rev. Drug Discovery 2009, 8, 579 10.1038/nrd2803. 19478820

[ref14] Kuang Y.; Balakrishnan K.; Gandhi V.; Peng X. Hydrogen Peroxide Inducible DNA Cross-Linking Agents: Targeted Anticancer Prodrugs. J. Am. Chem. Soc. 2011, 133, 19278–19281. 10.1021/ja2073824. 22035519 PMC3265938

[ref15] Saravanakumar G.; Kim J.; Kim W. J. Reactive-Oxygen-Species-Responsive Drug Delivery Systems: Promises and Challenges. Adv. Sci. 2017, 4, 1600124 10.1002/advs.201600124. PMC5238745 28105390

[ref16] Aluri S.; Janib S. M.; Mackay J. A. Environmentally Responsive Peptides as Anticancer Drug Carriers. Adv. Drug Delivery Rev. 2009, 61, 940–952. 10.1016/j.addr.2009.07.002. PMC2757494 19628014

[ref17] Gamcsik M. P.; Kasibhatla M. S.; Teeter S. D.; Colvin O. M. Glutathione Levels in Human Tumors. Biomarkers 2012, 17, 671–691. 10.3109/1354750X.2012.715672. 22900535 PMC3608468

[ref18] Oishi M.; Nagasaki Y. Stimuli-Responsive Smart Nanogels for Cancer Diagnostics and Therapy. Nanomedicine 2010, 5, 451–468. 10.2217/nnm.10.18. 20394537

[ref19] P R S.; James N. R.; P R A.; Raj D. K. Preparation, Characterization and Biological Evaluation of Curcumin Loaded Alginate Aldehyde–Gelatin Nanogels. Mater. Sci. Eng., C 2016, 68, 251–257. 10.1016/j.msec.2016.05.046. 27524019

[ref20] Oh J. K.; Drumright R.; Siegwart D. J.; Matyjaszewski K. The Development of Microgels/Nanogels for Drug Delivery Applications. Prog. Polym. Sci. 2008, 33, 448–477. 10.1016/j.progpolymsci.2008.01.002.

[ref21] Kabanov A. V.; Vinogradov S. V. Nanogels as Pharmaceutical Carriers: Finite Networks of Infinite Capabilities. Angew. Chem., Int. Ed. 2009, 48, 5418–5429. 10.1002/anie.200900441. PMC2872506 19562807

[ref22] Asadi H.; Khoee S. Dual Responsive Nanogels for Intracellular Doxorubicin Delivery. Int. J. Pharm. 2016, 511, 424–435. 10.1016/j.ijpharm.2016.07.037. 27444549

[ref23] Tahara Y.; Akiyoshi K. Current Advances in Self-Assembled Nanogel Delivery Systems for Immunotherapy. Adv. Drug Delivery Rev. 2015, 95, 65–76. 10.1016/j.addr.2015.10.004. 26482187

[ref24] Liu K.; Zheng D.; Zhao J.; Tao Y.; Wang Y.; He J.; Lei J.; Xi X. Ph-Sensitive Nanogels Based on the Electrostatic Self-Assembly of Radionuclide 131i Labeled Albumin and Carboxymethyl Cellulose for Synergistic Combined Chemo-Radioisotope Therapy of Cancer. J. Mater. Chem. B 2018, 6, 4738–4746. 10.1039/C8TB01295D. 32254301

[ref25] Landfester K. Miniemulsion Polymerization and the Structure of Polymer and Hybrid Nanoparticles. Angew. Chem., Int. Ed. 2009, 48, 4488–4507. 10.1002/anie.200900723. 19455531

[ref26] Dvořáková J.; Šálek P.; Korecká L.; Pavlova E.; Černoch P.; Janoušková O.; Koutníková B.; Proks V. Colloidally Stable Polypeptide-Based Nanogel: Study of Enzyme-Mediated Nanogelation in Inverse Miniemulsion. J. Appl. Polym. Sci. 2020, 137, 48725 10.1002/app.48725.

[ref27] Oehrl A.; Schötz S.; Haag R. Systematic Screening of Different Polyglycerin-Based Dienophile Macromonomers for Efficient Nanogel Formation through Iedda Inverse Nanoprecipitation. Macromol. Rapid Commun. 2020, 41, 1900510 10.1002/marc.201900510. 31750985

[ref28] Li S.; Zhang J.; Deng C.; Meng F.; Yu L.; Zhong Z. Redox-Sensitive and Intrinsically Fluorescent Photoclick Hyaluronic Acid Nanogels for Traceable and Targeted Delivery of Cytochrome C to Breast Tumor in Mice. ACS Appl. Mater. Interfaces 2016, 8, 21155–21162. 10.1021/acsami.6b05775. 27509045

[ref29] Farazi S.; Chen F.; Foster H.; Boquiren R.; McAlpine S. R.; Chapman R. Real Time Monitoring of Peptide Delivery in Vitro Using High Payload pH Responsive Nanogels. Polym. Chem. 2020, 11, 425–432. 10.1039/C9PY01120J.

[ref30] Devaraj N. K. The Future of Bioorthogonal Chemistry. ACS Cent. Sci. 2018, 4, 952–959. 10.1021/acscentsci.8b00251. 30159392 PMC6107859

[ref31] Herrmann A.; Kaufmann L.; Dey P.; Haag R.; Schedler U. Bioorthogonal in Situ Hydrogels Based on Polyether Polyols for New Biosensor Materials with High Sensitivity. ACS Appl. Mater. Interfaces 2018, 10, 11382–11390. 10.1021/acsami.8b01860. 29516719

[ref32] Fan L.; Lin C.; Zhao P.; Wen X.; Li G. An Injectable Bioorthogonal Dextran Hydrogel for Enhanced Chondrogenesis of Primary Stem Cells. Tissue Eng., Part C 2018, 24, 504–513. 10.1089/ten.tec.2018.0085. 30088443

[ref33] Koshy S. T.; Desai R. M.; Joly P.; Li J.; Bagrodia R. K.; Lewin S. A.; Joshi N. S.; Mooney D. J. Click-Crosslinked Injectable Gelatin Hydrogels. Adv. Healthcare Mater. 2016, 5, 541–547. 10.1002/adhm.201500757. PMC4849477 26806652

[ref34] Ramil C. P.; Lin Q. Bioorthogonal Chemistry: Strategies and Recent Developments. Chem. Commun. 2013, 49, 11007–11022. 10.1039/c3cc44272a. PMC3847904 24145483

[ref35] Malaprade L. Action of Polyalcohols on Periodic Acid and Alkaline Periodates. Bull. Soc. Chim. Fr. 1934, 1, 833–852.

[ref36] Mirgorodskaya O. A.; Poletaeva L. V. Periodate Oxidation of Dextrans. Pharm. Chem. J. 1985, 19, 347–351. 10.1007/BF00766342.

[ref37] Vercruysse K. P.; Marecak D. M.; Marecek J. F.; Prestwich G. D. Synthesis and in Vitro Degradation of New Polyvalent Hydrazide Cross-Linked Hydrogels of Hyaluronic Acid. Bioconjugate Chem. 1997, 8, 686–694. 10.1021/bc9701095. 9327132

[ref38] Alkanawati M. S.; da Costa Marques R.; Mailänder V.; Landfester K.; Thérien-Aubin H. Polysaccharide-Based pH-Responsive Nanocapsules Prepared with Bio-Orthogonal Chemistry and Their Use as Responsive Delivery Systems. Biomacromolecules 2020, 21, 2764–2771. 10.1021/acs.biomac.0c00492. 32530606 PMC7467571

[ref39] Kool E. T.; Park D.-H.; Crisalli P. Fast Hydrazone Reactants: Electronic and Acid/Base Effects Strongly Influence Rate at Biological Ph. J. Am. Chem. Soc. 2013, 135, 17663–17666. 10.1021/ja407407h. 24224646 PMC3874453

[ref40] Kölmel D. K.; Kool E. T. Oximes and Hydrazones in Bioconjugation: Mechanism and Catalysis. Chem. Rev. 2017, 117, 10358–10376. 10.1021/acs.chemrev.7b00090. 28640998 PMC5580355

[ref41] Bonam S. R.; Wang F.; Muller S. Lysosomes as a Therapeutic Target. Nat. Rev. Drug Discovery 2019, 18, 923–948. 10.1038/s41573-019-0036-1. 31477883 PMC7097195

[ref42] Wang R.; Dijkstra P. J.; Karperien M. Dextran. In Biomaterials from Nature for Advanced Devices and Therapies; John Wiley & Sons, 2016; pp 307–319.

