# Supplementary material for: Bio-Orthogonal Nanogels for Multiresponsive Release
Source: Biomacromolecules. 2021 Jun 15;22(7):2976–84. doi: 10.1021/acs.biomac.1c00378 (PMC8278386; doi:10.1021/acs.biomac.1c00378)
Supplement: Supplementary file 1 — bm1c00378_si_001.pdf [file bm1c00378_si_001.pdf]

Supporting Information

Bio-Orthogonal Nanogels for Multiresponsive Release

Mohammad Shafee Alkanawati,[a] *Marina Machtakova,*[a] *Katharina Landfester**[,a], *Héloïse Thérien-Aubin**[,a,b]

[a] Max Planck Institute for Polymer Research, Ackermannweg 10, 55128 Mainz, Germany

[b] Department of Chemistry, Memorial University of Newfoundland, 283 Prince Philip Dr, St. John's, NL, A1B 3X7, Canada

Experimental section

Materials

All chemicals and materials were used as received, if not otherwise mentioned. Polyglycerol polyricinoleate (PGPR) was obtained from Danisco and was purified first by dissolution in hexane followed by centrifugation (2000 rpm) to precipitate solid particles, then the supernatant was recover and dried to yield the purified PGPR.

Synthesis of nanocapsules precursors

Synthesis of functionalized dextran

The oxidation of the dextran was carried out by dissolving 5.0 g of dextran (M_n: 16 kDa, *Đ*: 2.18) and 2.75 g of KIO_4 in 200 mL of water. The resulting solution was stirred for 24 h at room temperature and then dialyzed (regenerated cellulose, MWCO =14 kDa) for 3 days against water (8 L, changed twice a day) and finally lyophilized, yielding the oxidized dextran in a quantitative manner (M_n: 16 kDa, *Đ*: 2.3).

The functionalization of the oxidized dextran with levulinic acid was carried out by the reaction of 3 g of oxidized dextran with 4.30 g of levulinic acid, 7.50 g of dicyclohexylcarbodiimide, 1.60 g of dimethylaminopyridine, and 1.50 g of pyridine in 100 mL of dry dimethylsulfoxide. The solution was reacted for 24 h at 60 °C under a nitrogen atmosphere and then dialyzed (regenerated cellulose, MWCO =14 kDa) over seven days against water (8 L, changed twice a day), and freeze-dried, yielding the levulinic acid-functionalized oxidized dextran in a quantitative manner (M_n: 18 kDa, *Đ*: 2.32).

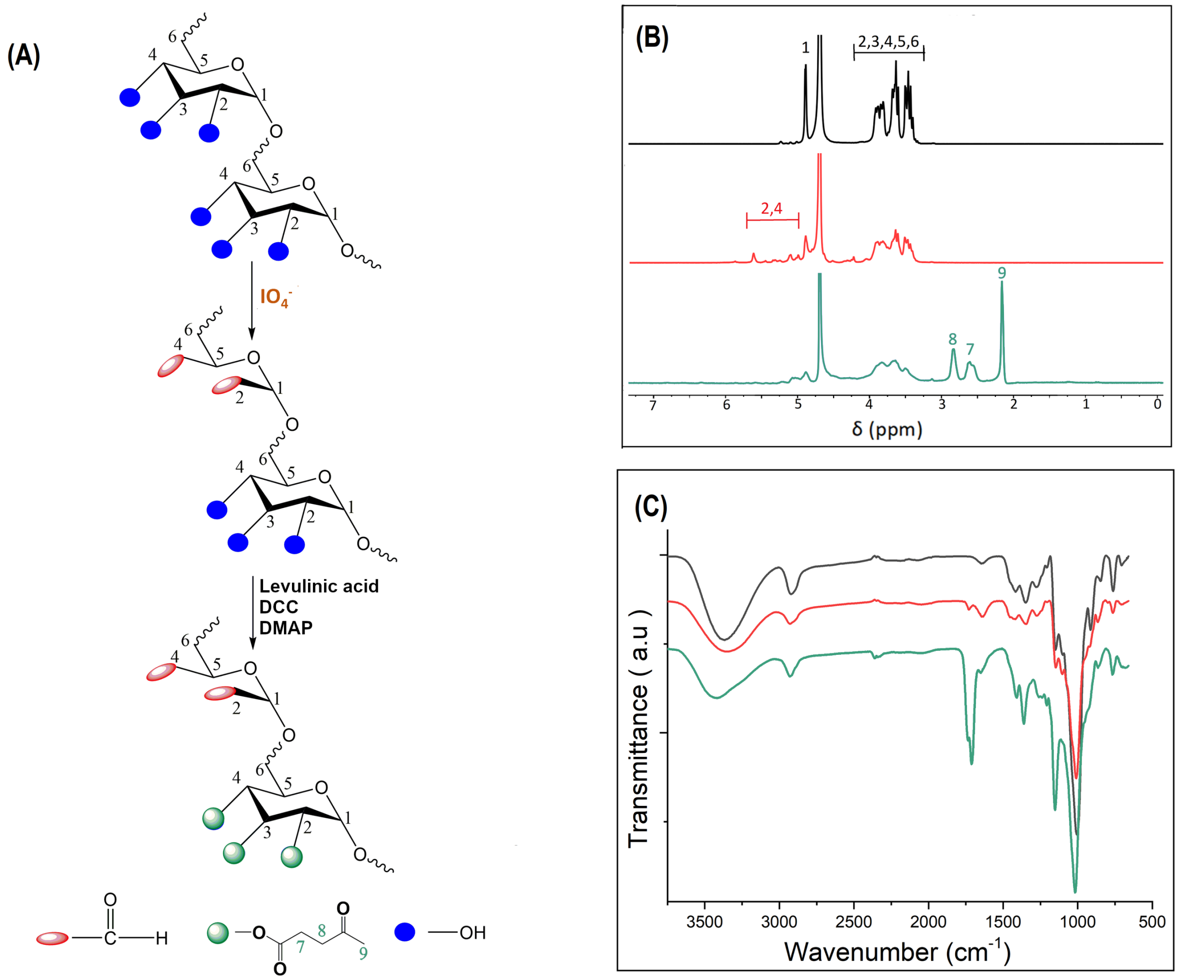

Figure S1. (A) Synthesis of the modified dextran. (B) ^{1}H-NMR spectrum of native dextran (black) and oxidized dextran (red) and final modified dextran (green). (C) FTIR spectrum of native dextran (black), oxidized dextran (red) and the final levulinic acid-functionalized oxidized dextran (green).

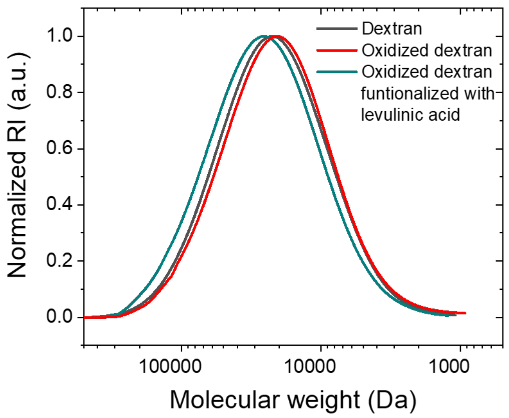

Figure S2. Molecular weight distribution of the dextran derivative measured by GPC in water, for native dextran (black), oxidized dextran (red) and the final levulinic acid-functionalized oxidized dextran (green).

The degree of oxidation of the dextran was measured before the functionalization with the levulinic acid by titration.[1] A sample of 0.1g of dextran was dissolved in 25 mL of a 0.25 N hydroxylamine hydrochloride solution in water. Each sample was reacted for 2 h at 50°C. The solution was then titrated with 0.1 M sodium hydroxide and methyl red. The change of the pH value with the addition of sodium hydroxide was recorded to determine the equivalent volume and compared to a blank sample prepared with unreacted dextran. The degree of functionalization with levulinic acid was calculated from the ratio of the NMR peak of the ketone protons of the levulinic acid and the proton on the C_1 of the dextran. On average, there were 0.75 aldehydes/glucose and 0.6 ketones/glucose in the modified dextran.

Synthesis of 3,3′-dithiodipropionic acid dihydrazide (DSNN)

In a 250 mL flask 3,3′-dithiodipropionic acid (5.00 g, 23.8 mmol) and ethanol (11.5 g, 480 mmol) were mixed with 50 g of toluene containing sulfuric acid (0.6 %). The resulting solution was refluxed for 15 hours at 110 °C. The reaction mixture was then cooled to room temperature, and the ethanol and toluene were evaporated under reduced pressure. The residue was then diluted with diethyl ether (60 mL), transferred to an extraction funnel, washed sequentially with saturated $NaHCO_3$ solution (30 mL) and water (30 mL). The organic phase was dried with $MgSO_4$ and evaporated under reduced pressure to afford crude dimethyl 3,3′-dithiodipropionate oil (5.21 g, 92 %). The resulting diethyl 3,3′-dithiodipropionate (5.00 g) was dissolved in methanol (100 mL) at room temperature. Hydrazine monohydrate (6.30 g, 6.0 eq.)

was then added and the reaction mixture stirred overnight (ca. 18 h) at room temperature. The resulting suspension was filtered and the white solid washed with methanol (50 mL), and then dried. The desired product, DSNN, was obtained as a white solid (3.10 g, 62%). Figure S3 shows the ^{1}H NMR spectra of the resulting DSNN in DMSO-d_6. NMR (300 MHz, DMSO-d6): δ = 9.05 (s, 2H; $NHNH_2$), δ = 4.20 (s, 4H; NH_2NH), δ = 2.88 (t, J = 7.2 Hz, 4H; CH_2CH_2S). δ = 2.40 (t, J = 7.2 Hz, 4H; CH_2SS).

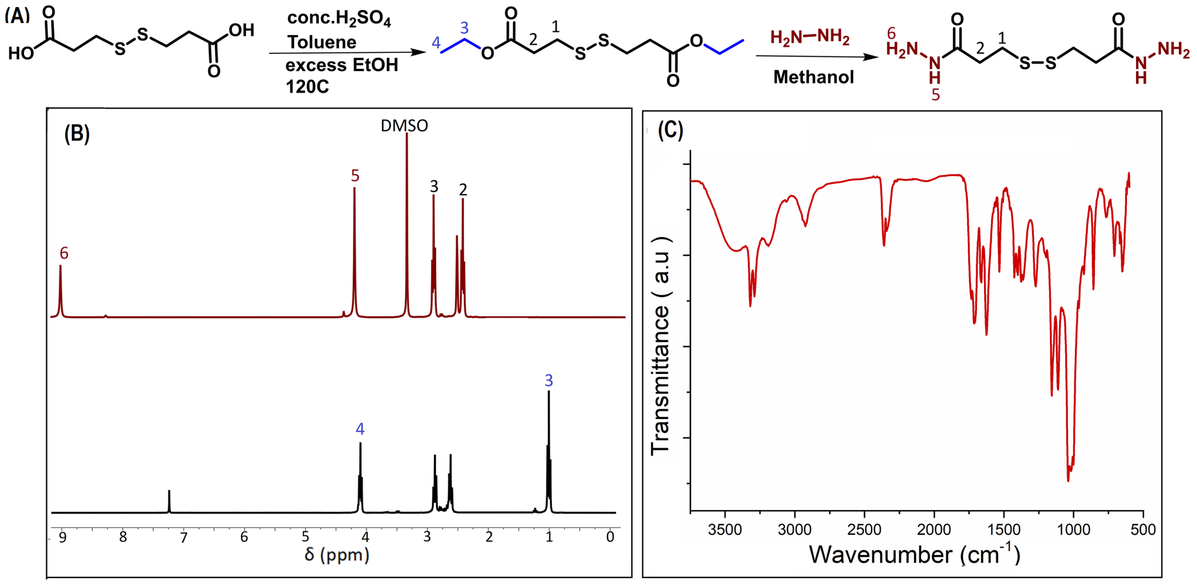

Figure S3. (A) Synthesis of 3,3′-dithiodipropionic acid dihydrazide (DSNN). (B) ^{1}H-NMR spectrum of DSNN (red) and dimethyl 3,3′-dithiodipropionate (black). (C) FTIR spectrum of DSNN.

Synthesis of thioketal dipropionic acid dihydrazide (TKNN)

In a 50 mL flask, butyl 3-mercaptopropionate (8.00 g) and anhydrous acetone (6.00 g) were mixed with a solution of hydrochloric acid in dioxane (HCl 4M, 4 mL). The resulting solution was stirred under nitrogen for 15 h at room temperature. The solvent was removed under reduced pressure. The residue was then diluted with diethyl ether (60 mL), transferred to an extraction funnel, washed sequentially with saturated $NaHCO_3$ solution (30 mL) and water (30 mL). The organic phase was dried with $MgSO_4$ and evaporated under reduced pressure to afford crude dibutyl thioketal dipropionate as a transparent, pale amber oil. The resulting dibutyl thioketal dipropionate (5.00 g) was dissolved in methanol (100 mL) at room temperature. Hydrazine monohydrate (6.30 g) was then added and the reaction mixture stirred overnight (ca. 18 h) at room temperature. The resulting reaction mixture was evaporated under

reduced pressure. The residue was then diluted with water (60 mL), transferred to an extraction funnel, washed twice with diethyl ether (30 mL). The aqueous phase was evaporated under reduced pressure to obtain the desired product as a white solid. Figure S4 shows the ^{1}H NMR spectrum of TKNN in DMSO-d6. NMR (300 MHz, DMSO-d6): δ = 9.05 (s, 2H; $NHNH_2$), δ = 4.20 (s, 4H; NH_2NH), δ = 2.88 (t, J = 7.2 Hz, 4H; CH_2CH_2S). δ = 2.40 (t, J = 7.2 Hz, 4H; CH_2S), δ = 1.5 (s, 6H; CH_3C).

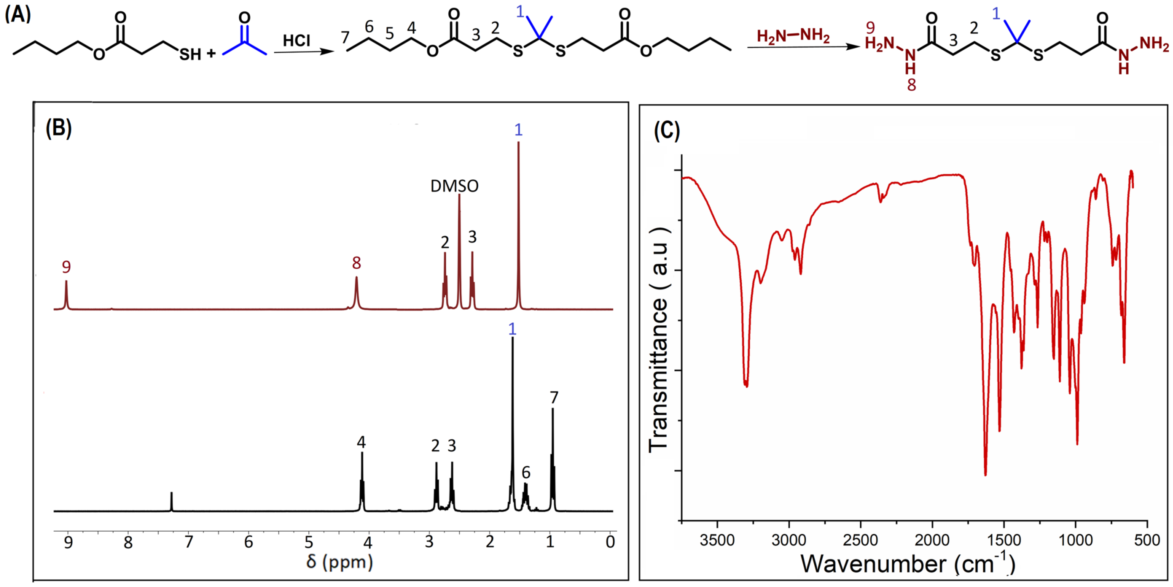

Figure S4. (A) Synthesis of thioketal dipropionic acid dihydrazide (TKNN). (B) ^{1}H-NMR spectrum of thioketal dipropionic acid dihydrazide (red) and dibutyl thioketal dipropionate (black). (C) FTIR spectrum of thioketal dipropionic acid dihydrazide.

Characterization

The hydrodynamic diameter and size distribution of the polymer nanogels were measured by dynamic light scattering (DLS) at 25 °C using a Malvern NanoS90, working at an angle of 90°. Equilibrated samples were first diluted with buffer at the appropriate pH value to a concentration of ca. 0.01 wt% of polymer and then analyzed. The Z-average hydrodynamic diameter and the polydispersity index (PDI) of the nanogels suspension were obtained from the Cumulant analysis of the DLS correlation curve. The surface zeta-potential of the nanogels in aqueous suspension was measure using a Malvern NanoZ. The samples for zeta-potential measurement were prepared from the same equilibrated suspension (pH=7.4) used for the DLS measurements; however, they were diluted to ca. 0.01 wt% using a solution of potassium chloride (1 mM). Morphological studies were performed by scanning electron microscopy (SEM) and transmission electron microscopy (TEM). For the sample preparation, one drop of

diluted nanocarrier suspension was placed onto a silica wafer (for SEM) or a carbon-coated grid (for TEM) and allowed to dry under ambient conditions. The SEM measurements were performed with a 1530 Gemini LEO field emission microscope (Zeiss), with an accelerating voltage of 170 V. For the TEM measurements, a Jeol 1400 transmission electron microscope was used with an accelerating voltage of 120 kV. FT-IR measurements were performed with the Perkin Elmer Spectrum BX FT-IR spectrometer and the spectra were recorded between 4000 and 600 cm^{-1}. For nuclear magnetic resonance (NMR) analysis, ^{1}H and ^{13}C NMR spectra were recorded with Bruker Avance spectrometers operating at a frequency of 300 MHz. Fluorescence intensity measurements were performed on an Infinite M1000 plate reader from Tecan using 96-well plates. The molecular weights of the polymers were measured by gel permeation chromatography (Agilent Technologies 1260 Infinity) equipped with a RI detectors (1260 RID) and calibrated either with dextran standards.

Quantification of the mixing efficiency during microfluidization.

Two droplet populations with a concentration of 10 wt% of dispersed phase were prepared independently by inverse miniemulsion in cyclohexane. The droplet population A was composed of a water solution containing 0.2 mg/mL of rhodamine and 0.2 mg/mL fluorescein in 0.01 M of HCl. The droplet population B contained a 0.01 M solution of NaOH. The ratio of fluorescence of the fluorescein and rhodamine mixture was affected by the local pH value within the droplet since the fluorescence of fluorescein decreases in acidic environments, while the fluorescence intensity of rhodamine remains unchanged across a broad pH range. When the dye solution was combined with the NaOH solution, the resulting titration of HCl by NaOH led to an increase in the fluorescence intensity of the fluorescein compared to the fluorescence of the rhodamine (Figure S5A). When 5 mL of each of the two corresponding emulsions were mixed inside the microfluidizer for 10 cycles, the same titration effect was observed. After every microfluidization cycle (896.3 bar) the fluorescence of the sample was measured. The results show that complete mixing between the acidic droplets containing the dye and the droplets containing the sodium hydroxide solution occurred after 3 or 4 cycles through the microfluidizer (Figure S5B).

HeLa cell culture

The HeLa cell line was cultivated with Dulbecco's Modified Eagle Medium (DMEM, Gibco/ThermoFisher, Germany) and supplemented with 10 % FBS, 1 % Glutamax, 100 U mL^{-1} penicillin, and 100 µg mL^{-1} streptomycin (all Gibco/Thermo Fisher, Germany).

Then, the cells were stored in an incubator (CO_2 Incubator C200, Labotect, Germany) at 37 °C, 5 % CO_2, and 95 % relative humidity for cultivation.

Viability and uptake experiments

The HeLa cell medium was removed and the cells were detached with 7 mL of 0.25 % Trypsin-EDTA (Gibco/Thermo Fisher, Germany) for 5 min at 37 °C, 5 % CO_2, and 95 % relative humidity. 7 mL of FBS supplemented medium was added to the cell suspension and the cells were centrifuged at 300 g for 5 min (5810R, Eppendorf, Germany). The supernatant was discarded and the cell pellet was resuspended in FBS supplemented medium. The cell viability and cell count were determined by mixing 20 µL of cell suspension with 20 µL of trypan blue followed by measurement with an automated cell counter (TC10, Bio-Rad, Germany).

Cell viability assay

After the cell harvesting, HeLa cells were seeded in a 96-well plate (white, item No.: 655083, GreinerBio-One, Austria) with a cell number of 5,000 cells per well. Nanogels dilutions in FBS supplemented DMEM (10 % FBS, 1 % Glutamax, 100 U mL^{-1} penicillin, and 100 µg mL^{-1} streptomycin) were prepared and added in a volume of 100 µL to the cells. Following overnight incubation at 37 °C and 5 % CO_2, the medium was removed. Samples were processed in triplicates. The cellTiter-Glo® Luminescent Cell Viability Assay (Promega, Germany) was performed according to the instructions of the manufacturer. The luminescence was measured with an Infinite M1000 plate reader (Tecan, Switzerland).

Cell uptake experiments

For the cell uptake experiments, HeLa cells were seeded in a 24-well plate after harvesting with a cell number of 100000 cells per well. Cells were incubated at 37 °C and 5 % CO_2 overnight to achieve the attachment. On the next day, the medium was removed and the cells washed once with 1 mL of cell medium. The nanocarriers were diluted with FBS supplemented DMEM (10 % FBS, 1 % Glutamax, 100 U mL^{-1} penicillin, and 100 µg mL^{-1} streptomycin) to a concentration of 75 µg mL^{-1} and added in a volume of 200 µL to the washed cells. Samples were processed in triplicates. Then, the HeLa cells were incubated at 37 °C and 5 % CO_2 for 2 h, and 24 h before measuring the cellular uptake. After the incubation of the cells with the nanogels, the nanogel suspension was removed and the cells were washed once with 1 mL of PBS. Then, the cells were detached by adding 250 µL 0.25% Trypsin-EDTA per well, and incubated for 5 min at 37 °C and 5 % CO_2. After incubation, 250 µL DMEM with FBS was

added and the detached cells were collected in 1.5 mL tubes. The cells were centrifuged at 300 g for 5 min and the supernatant was discarded. The cell pellet was resuspended in 1 mL of PBS. Further, flow cytometry measurements were conducted with an Attune™NxT (ThermoFisher, Germany) with a 638 nm excitation laser and a 670/14 nm band-pass filter. First, cells were analyzed with FSC/SSC to discriminate cell debris. Subsequently, the gated events of viable cells were analyzed by the fluorescent signal expressed as the median fluorescence intensity (MFI). Flow cytometry data analysis was conducted with Attune™NxT Software (Thermo Fisher, U.S.A.)

Additional results

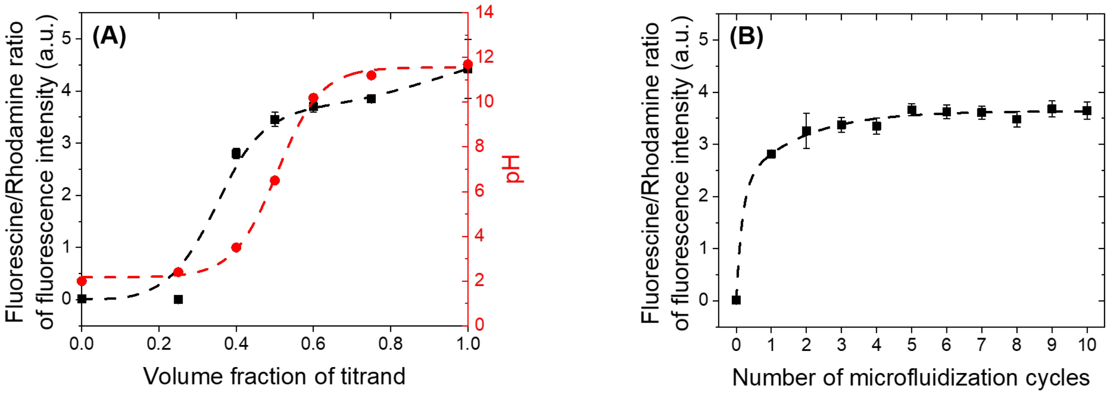

Figure S5. Evaluation of the efficiency of the droplet mixing during microfluidization. A) Variation of the ratio of the fluorescence intensity of fluorescein and rhodamine B after the macroscopic combination of a HCl containing dye solution titrated with a NaOH solution. B) Fluorescence intensity ratio between fluorescein and rhodamine B after the microfluidization of a mixture of droplets of HCl containing dye solution and droplets of NaOH solution.

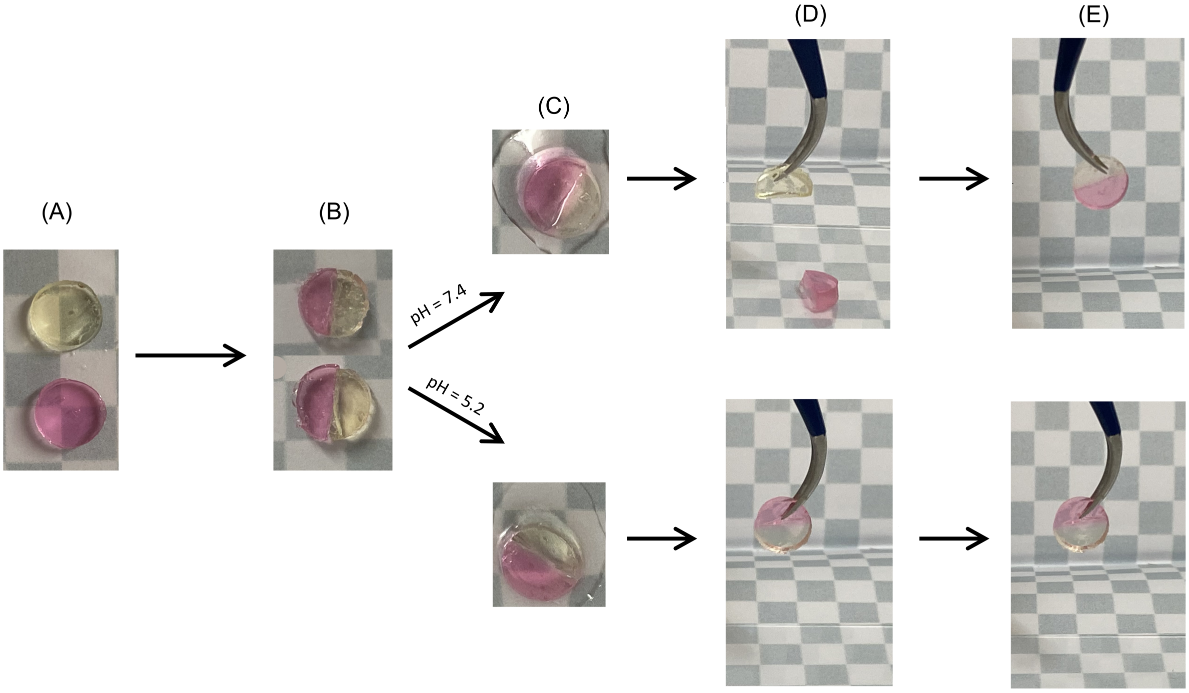

Figure S6. Preparation of macroscopic dynamic hydrazone network. A) Hydrazone gels prepared by the cross-linking of the modified dextran with adipic acid dihydrazide, with and without dye. B) The gel pieces were cut in half and brought back in close proximity. C) The pieces were immersed in solutions with different pH values (5.2 and 7.4). D) After 15 min the gel pieces in pH 5.2 had merged together, but those in pH 7.4 were still independent. E) However, after an extended contact period (24 h), the gel pieces were merged in both cases.

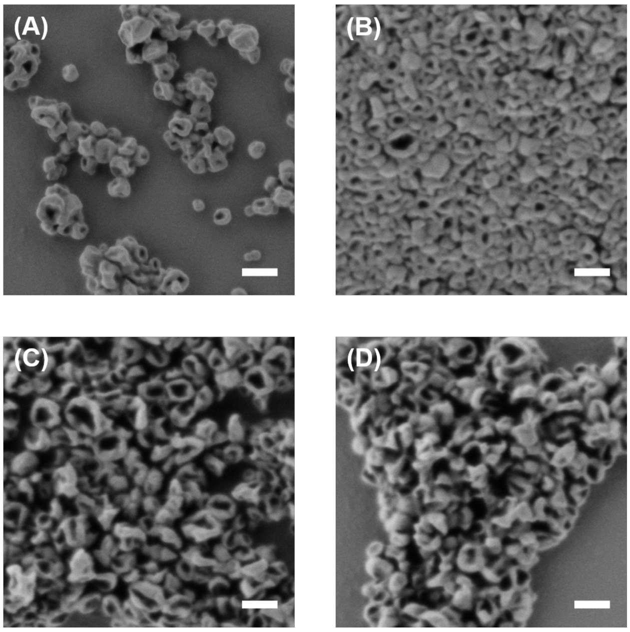

Figure S7. SEM image of the dextran nanogels prepared with (A) adipic acid dihydrazide (RNN), (B) 3,3'-dithiodipropionic acid dihydrazide (DSNN), (C) thioketal dipropionic acid dihydrazide (TKNN) or (D) a mixture of DSNN and TKNN. The scale bars are 250 nm.

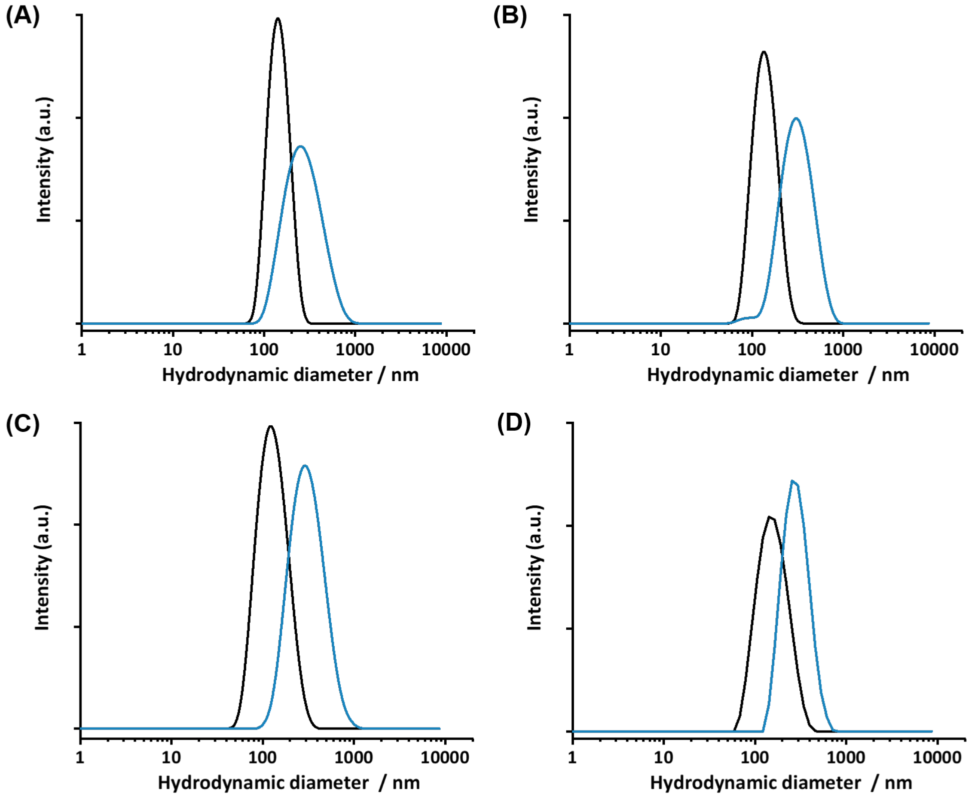

Figure S8. Distribution of the hydrodynamic diameter of the dextran nanogels prepared with (A) adipic acid dihydrazide (RNN), (B) 3,3'-dithiodipropionic acid dihydrazide (DSNN), (C) thioketal dipropionic acid dihydrazide (TKNN) or (D) a mixture of DSNN and TKNN, as prepared in cyclohexane (black) and after transfer in water (blue) as measured by DLS.

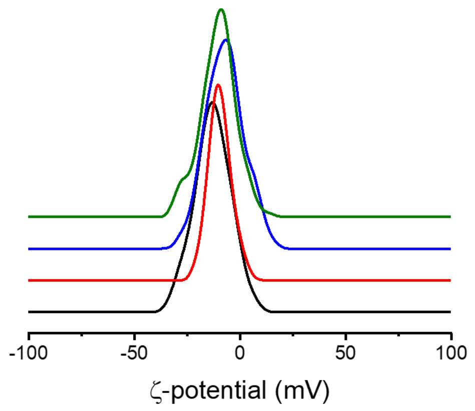

Figure S9. ζ-potential of the dextran nanogels prepared with adipic acid dihydrazide (RNN) (black), 3,3'-dithiodipropionic acid dihydrazide (DSNN) (red), thioketal dipropionic acid dihydrazide (TKNN) (blue) or a mixture of DSNN and TKNN (green).

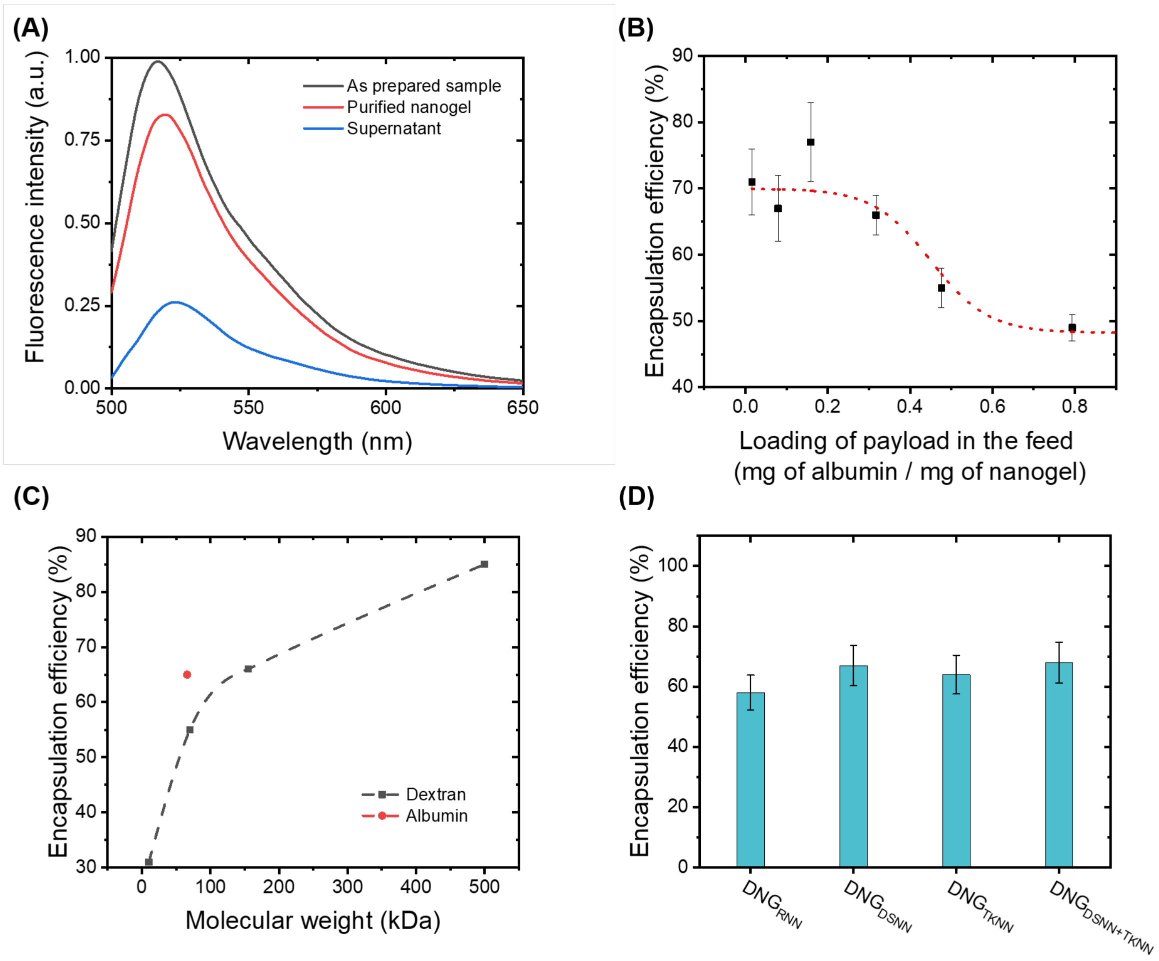

Figure S10. Encapsulation efficiency in the dextran nanogels. (A) Fluorescence emission spectra of FITC-albumin in the suspension of DNG following their transfer to water (Black), of the water separated from the DNGs by centrifugal filtration (Blue) and of the DNGs redispersed in PBS buffer (Red). (B) Effect of the loading of FITC-albumin in the precursor nanodroplets on the observed encapsulation efficiency. (C) Effect of the size of the payload on the encapsulation efficiency in the DNGs where FITC-albumin (red) is compared to a series of rhodamine-labeled dextran (black). (D) Effect of the cross-linker used to prepared the DNGs on the encapsulation efficiency measured for samples prepared with adipic acid dihydrazide (RNN), 3,3'-dithiodipropionic acid dihydrazide (DSNN), thioketal dipropionic acid dihydrazide (TKNN) or a mixture of DSNN and TKNN.

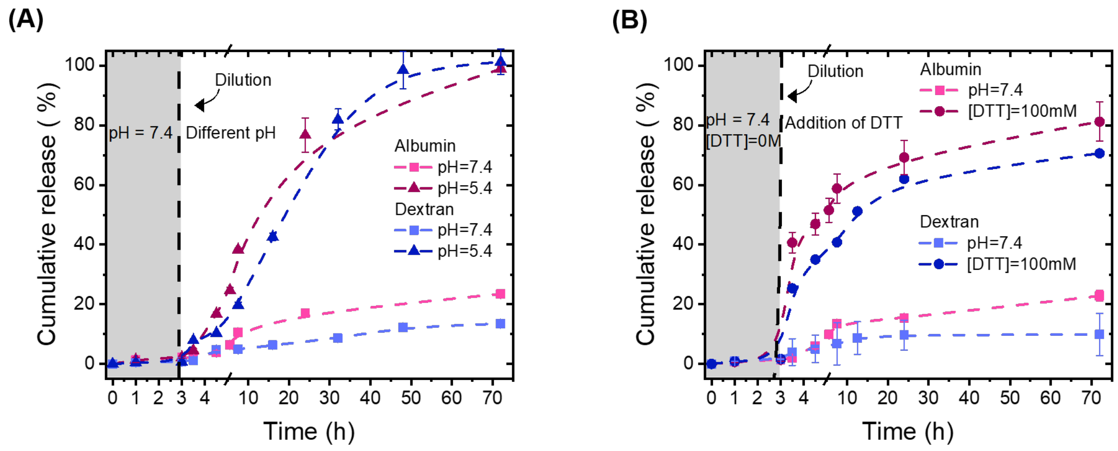

Figure S11. Release of tetramethylrhodamine-dextran (Blue) and FITC-albumin from (A) DNG_{RNN} at pH 7.4 (square) and pH 5.2 (triangle) and (B) DNG_{DSNN} at pH 7.4 without any DTT (square) and with 100 mM of DTT (circle).

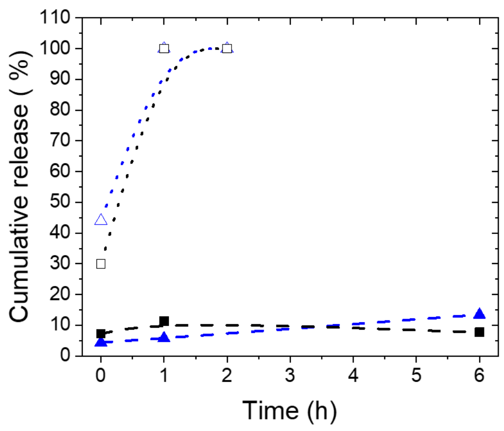

Figure S12. Release of tetramethylrhodamine-dextran from DNG_{RNN} at pH 7.4 (black) and pH 5.2 (blue). The DNGs were prepared with oxidized dextran with 1 aldehyde/glucose unit (solid symbol) or levulinate-dextran with 1.5 ketones/glucose unit (open symbol).

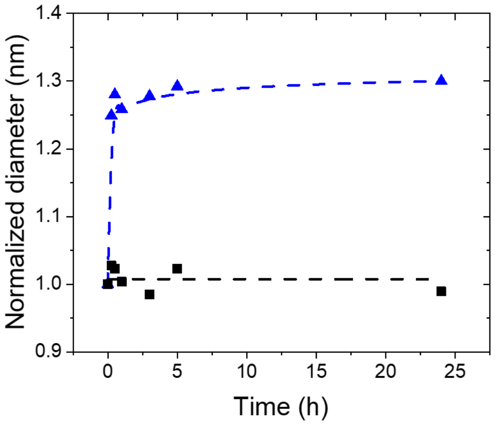

Figure S13. Effect of the pH value of the environment on the swelling of DNG_{RNN} after redispersion in buffer at pH 7.4 (black) and pH 5.2 (blue) from a suspension initially at pH 7.4.

Reference

1. Maltby, J. G.; Primavesi, G. R. The Estimation of Aldehydes, Ketones and Acetals by Means of the Hydroxylamine Hydrochloride Method. *Analyst* **1949,** 74 (882), 498-502
